# Workhouse Infirmary Management

**Published:** 1901-08-24

**Authors:** 


### WORKHOUSE INFIRMARY MANAGEMENT.

During the past thirty or more years a gradual process of evolution has been taking place in our system of poor-law administration, the effect of which has been to draw a broad line of distinction between the treatment of the sick and of the able bodied in our workhouses.

The poor laws are repressive laws, whose object is not the succouring of the poor but the repression of pauperism, and, this being the case, they are and they must be harsh and unbending, the very central idea in their administration being that the pauper who lives upon the rates shall never be better off than the labourer who maintains his independence by the sweat of his brow. That is and must be the controlling principle. But a softening influence has for years been eating its way into the administration of the poor law. It has been felt that age and sickness cannot be repressed, and that those whose fault is merely that they are old, or that they are ill, should be dealt with very differently from those who are obstinately idle; and in regard to the sick especially it has been felt that not only in the name of humanity, which would place the sick of all classes on a level, but even in that of mere political economy, which sees in many of these sick people the making of good workers if only they could be cured, we ought to do all we can for their comfort and their cure. Hence the Act of Parliament placing the metropolitan sick in specially built separate infirmaries; hence endless agencies for the alleviation of the miseries of the aged and infirm, and hence also the Workhouse Infirmary Nursing Association, which for over 21 years has devoted its attention to securing for our workhouse infirmaries all over the country some modicum at least of that skilled and careful nursing which the poor who go into our general hospitals receive as a matter of course. This Association has not only trained the nurses and supplied them to the workhouses, but has encouraged them to keep their places, and has done all it could to make it easy for the guardians to introduce trained nursing into their infirmaries.

At last, in August 1897, the great object of the Association seemed to be accomplished. For all these years it had endeavoured to persuade and to help the guardians to provide proper nursing for their sick. This had seemed proper work for a voluntary association, seeing that the guardians were under no legal compulsion to furnish such nursing. But in 1897 the Local Government Board issued an Order which, although far from perfect, did at least lay upon the guardians the obligation of providing paid nursing for their sick; absolutely prohibited all pauper nursing; demanded the employment of a certain small number of trained nurses under the name of superintendent nurses; and defined what sort of a training should be required for the latter post. Under these circumstances the days of persuasion seemed to be over, the Local Government Board had taken the matter up, and the Association retired.

We now find ourselves, however, face to face with a very grave condition of affairs, namely, that the law is being evaded, and that, although its evasion is known to the authorities, it is not being enforced. This is a most serious matter. If there is one point on which the British public has backed its opinion by solid cash it is in regard to the proper care of the sick poor. Hundreds of thousands of pounds are subscribed annually to the voluntary hospitals for the comfort and treatment and nursing of those sick who are not under the poor law; and in regard to those under the care of the guardians the public accepts the Orders of the Local Government Board as representing the manner of their treatment and takes for granted that up to that standard at least all is being done that should be done. What then are we to say when we find, as was shown by a deputation from the Workhouse Infirmary Nursing Association which waited upon Mr. Long on the 2nd of this month to be the case, that the Order has failed in providing a uniform standard of nursing; that the difficulty of obtaining and retaining trained women has increased; that regardless of the instructions of the Order, paupers are still largely employed in the capacity of nurses; that a large number of sham probationers are being employed and "trained" at places at which the means of training do not exist, probationers who doubtless at the end of this sham "training" will be turned out as certificated nurses; that the superintendent nurses are in many departments left under the control of the master and the untrained matron, and so hampered in their work; that, notwithstanding the Order, the disproportion still existing between the number of patients and of nurses involves "serious neglect of the sick by night"; and that the defects in the nursing "lead to frequent and regrettable cases of neglect, and sometimes to tragic consequences"? For most of these statements chapter and verse were given from the reports of Mr. Long's own inspectors, and we cannot but feel that an urgent case has been made out for a very material strengthening of the Order regulating the nursing in workhouse infirmaries. We are all the more driven to insist that something very definite should be done in this matter by comparing the state of affairs in England with that in Ireland. On this side of the water there has always been a very large leaven of good men among the guardians of the poor, men who have been anxious above all things to treat the sick kindly and well, and it is no doubt this leaven of good men that has held back, and that still holds back, the Local Government Board from issuing such a strong Order as would at once put all on a satisfactory footing. In Ireland, on the other hand—where the administration of the poor law by the local guardians has been a byeword—there, at last, the Local Government Board has screwed up its courage and issued a good strong Order. It would be curious, indeed, if it should turn out that the goodness of heart of the more advanced among the English guardians has really acted to the detriment of the sick in English workhouses by putting off reforms which otherwise might have been forced upon them.

